# Spatial epidemiological patterns suggest mechanisms of land-sea transmission for *Sarcocystis neurona* in a coastal marine mammal

**DOI:** 10.1038/s41598-020-60254-5

**Published:** 2020-02-28

**Authors:** Tristan L. Burgess, M. Tim Tinker, Melissa A. Miller, Woutrina A. Smith, James L. Bodkin, Michael J. Murray, Linda M. Nichol, Justin A. Saarinen, Shawn Larson, Joseph A. Tomoleoni, Patricia A. Conrad, Christine K. Johnson

**Affiliations:** 1 EpiCenter for Disease Dynamics, Karen C Drayer Wildlife Health Center, One Health Institute, University of California Davis, 1089 Veterinary Medicine Drive, Davis, CA 95616 USA; 2 U.S. Geological Survey, Western Ecological Research Center, Santa Cruz Field Station, 115 McAllister Way, Santa Cruz, CA 95060 USA; 3 Marine Wildlife Veterinary Care and Research Center, California Department of Fish and Wildlife, 151 McAllister Way, Santa Cruz, CA 95060 USA; 4 Department of Veterinary Medicine and Epidemiology, School of Veterinary Medicine, University of California Davis, 1089 Veterinary Medicine Drive, Davis, CA 95616 USA; 5 U.S. Geological Survey, Alaska Science Center, 4201 University Dr., Anchorage, AK 99503 USA; 6 Monterey Bay Aquarium, 886 Cannery Row, Monterey, CA 93940 USA; 7 Fisheries and Oceans Canada, Pacific Biological Station, 3190 Hammond Bay Road, Nanaimo, BC V9T 6N7 Canada; 8 New College of Florida 5800 Bay Shore Road, Sarasota, FL 34243 USA; 9 The Seattle Aquarium, 1483 Alaskan Way, Pier 59, Seattle, WA 98101 USA; 10 Department of Pathology, Microbiology and Immunology, School of Veterinary Medicine, University of California Davis, 1089 Veterinary Medicine Drive, Davis, CA 95616 USA; 11 Present Address: Acadia Wildlife Services, P.O. Box 56, South Freeport, ME 04078 USA; 12 Present Address: Nhydra Ecological Consulting, 11 Parklea Dr Head of St, Margarets Bay, NS B3Z2G6 Canada

**Keywords:** Ecological epidemiology, Risk factors

## Abstract

*Sarcocystis neurona* was recognised as an important cause of mortality in southern sea otters (*Enhydra lutris nereis*) after an outbreak in April 2004 and has since been detected in many marine mammal species in the Northeast Pacific Ocean. Risk of *S. neurona* exposure in sea otters is associated with consumption of clams and soft-sediment prey and is temporally associated with runoff events. We examined the spatial distribution of *S. neurona* exposure risk based on serum antibody testing and assessed risk factors for exposure in animals from California, Washington, British Columbia and Alaska. Significant spatial clustering of seropositive animals was observed in California and Washington, compared with British Columbia and Alaska. Adult males were at greatest risk for exposure to *S. neurona*, and there were strong associations with terrestrial features (wetlands, cropland, high human housing-unit density). In California, habitats containing soft sediment exhibited greater risk than hard substrate or kelp beds. Consuming a diet rich in clams was also associated with increased exposure risk. These findings suggest a transmission pathway analogous to that described for *Toxoplasma gondii*, with infectious stages traveling in freshwater runoff and being concentrated in particular locations by marine habitat features, ocean physical processes, and invertebrate bioconcentration.

## Introduction

*Sarcocystis neurona*, most widely known as the main etiologic agent of equine protozoal myeloencephalitis, is one of two pathogens that causes fatal protozoal encephalitis (PE) in sea otters (*Enhydra lutris*). Following recognition of this disease in sea otters^1^, *S. neurona* and *Toxoplasma gondii* were identified as important causes of mortality of southern sea otters (*E. lutris nereis*)^1^. Although exposure to *S. neurona* may be slightly less common overall^2^, *S. neurona*-related disease is generally more severe, at least in acute and subacute cases^1,2^. A mass-stranding event in 2004 was attributed largely to *S. neurona* infection: forty sick or dead sea otters stranded on beaches near Morro Bay, California, USA within one month of each other, and this parasite was found to be a primary or contributing cause of death in 15 of 16 animals submitted for full necropsies during this event^3^.

**Figure 1:**
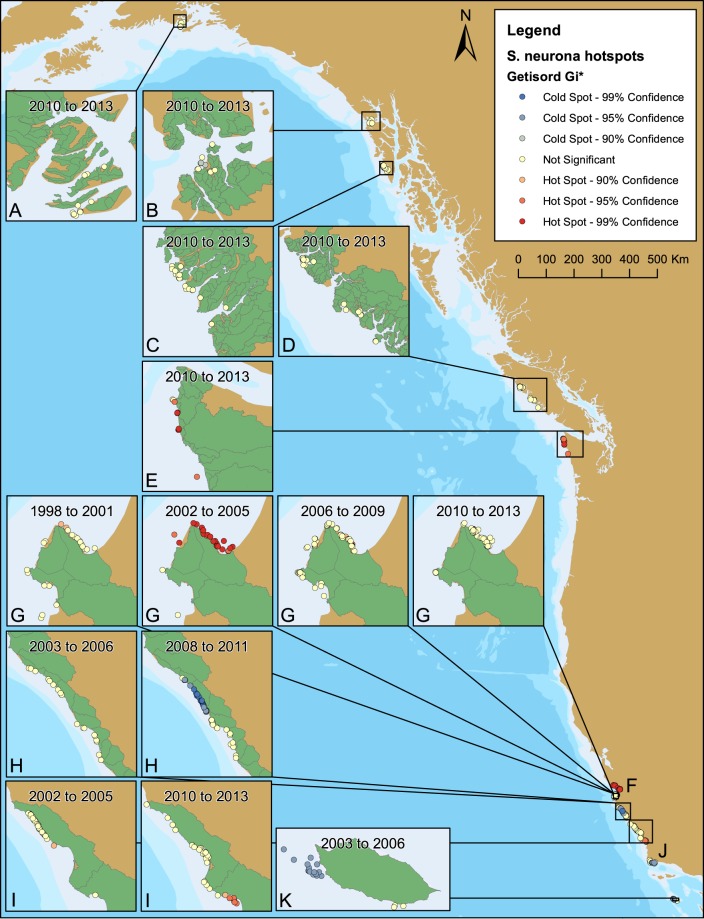
Map of all study sites. **A** = Western Prince William Sound, Alaska, USA; **B** = Elfin Cove, Alaska, USA; **C** = Whale Bay, Alaska, USA; **D** = Nuchatlitz Inlet & Clayoquot Sound, British Columbia, Canada; **E** = Olympic Peninsula, Washington, USA; **F** = Monterey Bay, California USA; **G** = Monterey Peninsula, California, USA; **H** = Big Sur, California, USA; **I** = San Luis Obispo, California, USA; **J** = Santa Barbara Channel, California, USA; **K** = San Nicolas Island, California, USA. Sea otters (n = 711) were captured for this study between 1998 and 2013. Coloured circles show capture locations of animals coded according to the space-time hotspot analysis of sea otter *Sarcocystis neurona* serum indirect fluorescent antibody test (IFAT) results from live-captured sea otters (n = 711). P-values are calculated using the Getis-Ord Gi statistic^16^. This statistic is primarily a local space-time comparison, and so it is not informative to compare colors between regions (i.e. low-prevalence study sites do not appear uniformly blue). A region with consistently low prevalence across space and time appears yellow. Map created using ArcGIS version 10.7.1 (ESRI https://www.esri.com/en-us/arcgis/about-arcgis/overview).

*Sarcocystis* spp. have an obligatory two-host lifecycle. The intermediate host range of *S. neurona* is broader than most species of this genus and includes both terrestrial and marine mammal species^4^. The only known definitive (sporocyst-shedding) host of *S. neurona* in North America is the Virginia opossum (*Didelphis virginiana*), and sporocysts remain viable in the environment for months. This parasite also infects and forms tissue cysts in a wide variety of endothermic intermediate hosts^4^. However, because sea otters prey almost exclusively on ectotherms, ingestion of tissue cysts by consumption of warm-blooded intermediate hosts is not likely, and rather ingestion of sporocysts^2^, either directly or indirectly (consuming a transport host, such as a filter-feeding invertebrate), is the most plausible route of exposure^5^. *Sarcocystis neurona*-associated sea otter deaths in California peak in the spring/early summer, and are temporally associated with large rainfall events^2,6^. Freshwater outflows peak in late winter and early spring in this region, supporting the present theory of land-sea transmission of *S. neurona* as a pathogen pollutant. This land-sea transmission pathway is also consistent with molecular evidence that terrestrial and marine *S. neurona* isolates are not genetically distinct^7^. Though hotspots of high *S. neurona* seroprevalence have been identified^8^, it is not known whether specific features of the terrestrial environment enhance infection risk, either due to high loadings of infectious sporocysts onto the landscape or due to enhanced runoff and transfer of sporocysts into the marine food-web. Areas of higher than average *S. neurona* seroprevalence in sea otters have been identified in areas with abundant soft-sediment substrate such as Estero Bay (see Fig. 1 for study site locations) and southern Monterey Bay^8^, suggesting a role for bivalves in promoting transmission to sea otters. A dietary preference for clams and other soft sediment prey items has been described as a strong risk factor for *S. neurona* exposure^8^. A mechanism to explain this association has not been experimentally demonstrated, but direct ingestion of sporocysts by bivalves during filter-feeding is plausible - bivalves have been experimentally shown to concentrate and retain free-floating *Toxoplasma gondii* oocysts and *Giardia* spp. cysts from water^9^.

Most research on *S. neurona* in sea otters has focused on California, but the range of the Virginia opossum extends north as far as Washington state and the lower Fraser valley in British Columbia. Examination of stranded animals in the Pacific Northwest has revealed that *S. neurona*-associated meningoencephalitis and myelitis is also a significant cause of marine mammal mortality outside of California. A study including 161 animals (both pinnipeds and cetaceans) from the Pacific Northwest showed *S. neurona* (tested by PCR) to be a common cause of mortality: PE was found to be the primary cause of death in 25% (7/28) of animals infected with *S. neurona* alone and 44% (22/50) of *S. neurona/T. gondii* co-infected animals^10^. A more recently published survey of stranding mortalities among marine mammals in the northwestern USA and southwestern Canada found 60% (136/227) of pinniped and cetacean carcasses with signs consistent with PE tested positive for *S. neurona* by Polymerase Chain Reaction (PCR)^11^.

Based on the assumption that *S. neurona* sporocysts shed by definitive hosts could reach sea otters via terrestrial runoff in a way similar to other environmentally-resistant fecal stages of related apicomplexan parasites, we hypothesise that exposure risk in this host species is associated with terrestrial landscapes that either support higher opossum population density, generate increased runoff due to hydrological characteristics (including development activities and watershed modification), or a combination of both factors. In this study we assess the characteristic features of areas of heightened risk of *S. neurona* exposure, while also accounting for individual and behavioral risk factors using a large sample of live captured sea otters from California, Washington, British Columbia and Alaska collected between 1998 and 2013.

## Materials and Methods

### Sample collection

Data for this study were collected in 13 study regions in Alaska (AK), Washington (WA) and California (CA). USA and British Columbia (BC), Canada between 1998–2013 (Fig. 1, Table S1). Data collection occurred from 1998–2013 in CA and 2010–2013 in WA, BC and AK. See Table S2 for details) Sea otters (n = 711) were captured by experienced divers using Wilson traps or with tangle nets^12^. All animals were anesthetised using fentanyl (0.33 mg/kg) and midazolam (0.11 mg/kg) by intramuscular injection^13^ to facilitate physical examination. Weight, length and sex were recorded, and animals were classified into three age classes based on estimates of tooth wear - juvenile (0–1.5 yr), subadult (1.5–3 yr) and adult (>3 yr). Blood samples were collected by jugular venipuncture and centrifuged at 1500 x g for 10 min. Serum was separated and stored at −70 °C until testing. A subset (n = 131) of apparently healthy, non-palpably pregnant animals received surgically implanted VHF radios and time-depth recorders to facilitate tracking and behavioral observations.

### Laboratory analysis and estimating probability of infection

Serum samples were tested for *S. neurona* antibodies using an indirect fluorescent antibody test validated in horses (IFAT)^14^. As this assay has not been validated for detection of *S. neurona* infection in sea otters, a cutoff was selected by use of a two-component log-normal mixture model. The model was fitted to the results from all live-captured sea otter serum samples that have been tested using this assay and a similarly specified mixture model was fitted to all IFAT results for the related parasite *T. gondii*, a similar test with a previously validated cutoff of 1:320^15^. Results are expressed as titers, representing the maximum dilution of serum which yields a positive result. A cutoff dilution value was selected to ensure a similar posterior probability for *S. neurona* as obtained for the validated *T. gondii* test. Where animals were sampled multiple times, an individual was classified as positive if it had tested positive at any time. For all data other than IFAT, data from the most recent capture were used.

### Space-time cluster analysis

In order to describe the spatiotemporal distribution of cases, a local hotspot analysis was performed including all sea otter *S. neurona* IFAT results from 1998–2013, including samples from otters that were recaptured (total n = 972). The Getis-Ord Gi* statistic^16^ was calculated with a 4 year time window using ArcGIS 10.3 (ESRI, Redlands, California, USA). Statistically significant hotspots were identified using Z-scores at confidence levels of 90%, 95% and 99%.

### Geospatial data

All watersheds in the study area were delineated, outflow points were defined and geospatial variables were calculated according to the methods described previously^17^. In the absence of specific data on population density of the definitive host (Virginia opossum), indices of land use, land cover and human population density were examined for associations with increased or decreased *S. neurona* seroprevalence, as all of these variables may be associated with opossum density or the probability of sporocysts shed by opossums being transferred to the ocean in runoff. Briefly, values of human population density, housing unit density, road density and proportion of each land use class were calculated for each watershed with ArcGIS 10.3 (ESRI, Redlands, California) and Geospatial Modeling Environment (0.7.3.0)^18^ using data from the U.S. census^19^, census of Canada^20^, the National Land Cover Database^21^ and North American Land Cover Database^22^. Also following the methods employed previously^17^, the exposure weighting (W_i,j_) for sea otter *i* to watershed *j* was calculated as the quotient of *Q*_*j*_ *-* the mean annual discharge of water (m^3^s^−1^) from watershed j; and *D*_*ij*_ - the distance between capture location and the outflow point of the watershed:

$${W}_{i,j}=\frac{{Q}_{j}}{{D}_{ij}}$$

A cutoff distance of 100 km was applied, assuming no contact with watersheds more than 100 km from the capture location. In northern sea otter study sites, the shortest effective distance between an otter capture location and a pour point (location where a river meets the coastline), excluding land barriers, was found using ‘gdistance’ in R^23^ and distance along this path was calculated using the package ‘sp’ with all points projected in meters (UTM). Distances in CA were calculated using ‘as the otter swims’ (ATOS) distances, meaning the distance along the 10 m depth contour, since the coastline of California is essentially linear and for some earlier captures, location was recorded only as a location along the ATOS line to the nearest 0.5 km. As a number of land use/land cover variables and census-derived measures are highly correlated, such as population density, housing unit density and road density, an eigenvector decomposition with varimax rotation was performed using the package ‘psych’ in R. Five orthogonal components (Table S3) describing the underlying variation in these measures were derived and scores for each were calculated for all study animals.

### Marine habitat analysis

For California sea otters (n = 535), an analysis of marine substrate and habitat was conducted. Percentage cover of soft and hard substrate types was calculated for coastal polygons representing potential sea otter habitat defined by the 0 m and 10 m isobaths divided in 0.5 km segments along the California coastline (Seafloor data were acquired 2006–2009, processed and archived by the California Seafloor Mapping Project, a partnership between the Seafloor Mapping Lab at California State University, Monterey Bay; Fugro Pelagos Inc.; U.S. Geological Survey, Western Coastal & Marine Geology; and Moss Landing Marine Lab, Center for Habitat Studies). Kelp cover was estimated from satellite images for the same polygons (Kelp canopy resource data were collected, analysed and distributed by the California Department of Fish and Wildlife 2002–2006). Exposure values for individual sea otters to soft, hard, and mixed substrate cover as well as kelp cover were calculated as an inverse distance-weighted average of all polygons within 100 km ATOS of the capture location:

$${W}_{i,j}=\frac{1}{{D}_{ij}}$$

Individual exposure to substrate type was examined both as continuous variables (proportional cover of soft sediment, hard substrate, kelp cover) and as a categorical variable with levels of hard (≥60% hard substrate), soft (<10% hard substrate) and mixed substrate (>10% but <60% hard substrate).

### Diet composition

Diet composition was assessed for the tagged animals (n = 131) using previously described methods^24,25^. Experienced shore- or boat-based observers recorded data during sea otter foraging bouts using spotting scopes. Prey items were classified into 24 functional taxonomic/morphological groups and biomass was estimated based on the count and size of prey items^26^. Previous work has found that diet data is repeatable over an extended period of time as sea otters develop pronounced diet specialization, particularly in areas of high population density^25^. Only individuals with at least 10 foraging bouts and 300 recorded dives were included in analyses. Animals representing the entire distribution of sea otters in California were included in this analysis.

### Risk factor analysis

Marginal associations of individual (age, sex, diet) and spatially calculated variables (land cover indices, kelp cover, substrate type) with *S. neurona* antibody status were examined using univariate logistic regression. Multivariate mixed effects logistic regression models were fit using hypothesis driven combinations of these variables and a random effect for study region (n = 13) to account for correlation of observations within discrete study regions. Three final multivariate models are reported: i) a model including all animals from all study regions with variables selected using a backwards stepwise method, ii) a subset analysis including all animals captured in California, including variables describing the marine substrate in potential sea otter habitat within 100 km of the capture location, and iii) a subset analysis including only animals with quantitative diet analysis data (also limited to California sea otters). Selection for models ii) and iii) started from the same formulation as in i) with the addition of either habitat or diet variables. Non-significant variables were removed in a backward stepwise fashion based on likelihood ratio tests. Fit of non-nested models was compared using the Akaike Information Criterion (AIC) and R^2^ values^27^.

#### Ethics statement

All animal care and use protocols were evaluated and approved by the Institutional Animal Care and Use Committee at the University of California Santa Cruz (approval no. Tinkt1306) and all experimental methods were performed in accordance with the relevant guidelines and regulations. Any use of trade, product, or firm names in this publication is for descriptive purposes only and does not imply endorsement by the U.S. government. Wild animal work was conducted under U.S. Fish and Wildlife permits PRT-766818 (Bodkin) and MA672624 (Tinker).

## Results

Overall 22.4% (159/711) of sea otters tested positive for *S. neurona*, and we detected marked spatial variation in *S. neurona* seroprevalence along the eastern Pacific Rim. Higher *S. neurona* seroprevalence was associated with increasing age class, male sex and diets high in clams (Table 1). The highest seroprevalence was found in animals living in habitats with low kelp cover and close to densely populated or developed areas, cropping land and wetlands.

**Table 1:** Multivariate logistic regression models predicting exposure to *Sarcocystis neurona* in live captured sea otters (1998–2013).

Variable	Level	OR	95% CI	P-value
**Panel 1**				
**Sex**	Female	1.00	—	REF
	**Male**	**2.94**	**(1.87–4.6)**	**<0.001**
**Age**	Juvenile	1.00	—	REF
	Subadult	2.54	(0.42–15.44)	**0.311**
	**Adult**	**13.96**	**(3.15–61.92)**	**<0.001**
**C1 (Developed/Row Crops)**	**1 unit increase**	**1.65**	**(1.35–2.01)**	**0.001**
**C4 (Wetlands)**	**1 unit increase**	**2.56**	**(2.04–3.22)**	**<0.001**
				**R**^**2**^ **= 0.174**
**Panel 2**				
**Sex**	Female	1.00	—	REF
	**Male**	**2.46**	**(1.52–3.99)**	**<0.001**
**Age**	Juvenile	1.00	—	REF
	Subadult	2.17	(0.35–13.44)	**0.406**
	**Adult**	**13.02**	**(2.93–57.83)**	**<0.001**
**C1 (Developed/Row Crops)**	**1 unit increase**	**1.48**	**(1.19–1.84)**	**<0.001**
**C4 (Wetlands)**	**1 unit increase**	**2.48**	**(1.73–3.55)**	**<0.001**
**Kelp Cover**	**1% increase**	**0.98**	**(0.96–1)**	**0.037**
				**R**^**2**^ **= 0.134**
**Panel 3**				
**Sex**	Female	1.00	—	REF
	**Male**	**7.54**	**(2.18–26.09)**	**0.001**
**C4 (Wetlands)**	**1 unit increase**	**13.94**	**(2.81–69.18)**	**0.001**
**Clam**	**10% increase**	**1.59**	**(1.13–2.23)**	**0.008**
				**R**^**2**^ = **0.168**

Each panel denotes a logistic regression model based on different datasets. *Panel 1:* Includes all study animals (n = 711) from Alaska, British Columbia, Washington and California. *Panel 2:* Includes only animals captured in California where detailed habitat data were available (n = 535). *Panel 3:* Includes only animals captured in California where detailed habitat and individual diet data were available (n = 131). Kelp area refers to the weighted (by distance) individual exposure to kelp coverage within nearby (<100 km from capture location) potential sea otter habitat. C1 and C4 are respectively the 1^st^ and 4^th^ orthogonal components of an eigenvector decomposition analysis including all landuse/landcover variables and the three census-derived variables (population density, housing unit density and road density). C1 is strongly associated with high population density, developed area and row crops. C4 is strongly associated with wetland area (see Supplementary Analyses for further details). OR = Odds ratio. 95% CI = 95% confidence interval (wald). P-values were calculated by the wald method. R^2^ values were calculated according to the method described by Jaeger *et al*. (2017).

Seroprevalence ranged from 0% at Clayoquot Sound, British Columbia and San Nicolas Island, California to 82% in Monterey Bay, California (Table S2). High seroprevalence (67%) was also recorded at Elkhorn Slough, California and Olympic Peninsula, Washington. Very low prevalences (10% or less) were recorded in Alaska (Western Prince William Sound, Whale Bay and Elfin Cove), and no titers above 1:320 were recorded at these sites. Nuchatlitz Inlet, British Columbia however, yielded a prevalence of 13% with titers as high as 1:5120.

Hotspot analysis (Fig. 1) identified clusters of high *S. neurona* seroprevalence in Monterey Bay, CA and Elkhorn Slough, CA from 1998–2005 and 2010–2013, at Olympic Peninsula, WA in 2011 and at San Luis Obispo, CA from 2010–2013. Low seroprevalence was recorded at San Nicolas Island from 2001–2004, at Big Sur from 2008–2011 and in the Santa Barbara Channel from 2010–2013.

The best fit regression model for the entire study population (n = 711; Table 1, panel 1 – see also Table S4 for univariate results) showed a positive association between *S. neurona* serum antibody status and male sex (OR = 2.9, p < 0.001). Adults also had much higher exposure odds than juveniles and subadults (OR = 14.0, p < 0.001). High scores for land use/land cover components C1 (human population density, developed area and row crops) and C4 (wetlands) were both strongly associated (p ≤ 0.001) with increased *S. neurona* seroprevalence. The California-only subgroup (n = 535) analysis including marine substrate type (Table 1, panel 2) showed a similar increase in *S. neurona* seroprevalence risk with age (OR = 13.0 for adults compared with juveniles and pups, p < 0.001) and male sex (OR = 2.5 compared with females, p < 0.001). High scores for land use/land cover components C1 and C4 were again strongly associated (p < 0.001) with increased *S. neurona* seroprevalence. In this analysis, increased seroprevalence risk was also associated with decreasing kelp area (OR = 0.98 per 1% increase in kelp cover, p = 0.037). Though marine substrate type was predictive of antibody status when kelp cover was not considered, it did not enhance predictive value in addition to kelp cover and the model including kelp cover as the only marine habitat variable fit the data better based on AIC values. A third model fitted to the subset of animals (n = 131) with diet data (Table 1, panel 3; see also Table S5) found male sex (p = 0.001), proximity to watersheds containing wetlands (p = 0.001) and consumption of a diet containing a greater percentage of clams by biomass (OR = 1.6 per 10% increase, p = 0.008) to be significant predictors of *S. neurona* seroprevalence.

## Discussion

We examined broad-scale descriptors of coastal watersheds, local marine habitat characteristics and individual animal diet preferences, and found that each is correlated with sea otter *S. neurona* seroprevalence risk. The highest *S. neurona* seroprevalence risk was found in animals living in habitats with low kelp cover and adjacent to developed lands, areas of denser human settlement, cropping land and wetlands. Within California, the relationship of *S. neurona* prevalence with human population density and land use remained statistically significant even after accounting for variation in marine habitat/substrate, sex, and diet.

In agreement with earlier research on the California sea otter population^8^, male animals and older age classes demonstrated markedly higher seroprevalence, as did animals with at least 10% clams in their diet. Also re-affirmed in this larger dataset was the association between soft-sediment marine habitat and high *S. neurona* prevalence^8^. All high seroprevalence areas in the present study were characterised by soft sediment habitat, and many were located near the mouths of large rivers or estuaries. Conversely animals using kelp-dominated habitat with a preponderance of rocky substrate at Big Sur, Santa Barbara Channel exhibited average or below-average seroprevalence (Table S2). Outside the period from 2002–2005 (including the 2004 outbreak – Table S2), the rocky Monterey Peninsula area also exhibited low seroprevalence. Consumption of high-risk prey (clams) that predominate in soft sediment habitat most likely explains this relationship, though terrestrial factors could also be synergistic. Furthermore, within California, low *S. neurona* seroprevalence was observed among sea otters living in areas with high kelp cover. This association is likely not due to a protective effect of kelp, but rather as an indicator of substrate/habitat type - high kelp cover does not occur in predominantly soft substrate areas. These findings suggest a role for clams or other prey living in soft sediment habitats in concentrating *S. neurona* sporocysts, thereby increasing the likelihood of ingestion by a sea otter.

Invertebrate bioconcentration of *S. neurona* sporocysts by clams or other bivalves has biological precedent. An analogous but distinct process is believed to occur with *T. gondii* oocysts, mediated by aggregation in marine polymers, adhesion to kelp blades and ingestion by turban snails (*Tegula spp*.)^28–30^. Our findings suggest that a similar process might occur with this related apicomplexan parasite, but in soft sediment habitats, rather than in kelp forests, and with a resident bivalve rather than a gastropod. This difference may be attributable to differences in the surface properties, aggregation and movement of the *S. neurona* sporocysts compared to *T. gondii* oocysts in estuarine and marine environments, or may simply reflect the differing features of locations where large numbers of the respective species of protozoa are discharged into the ocean. Further study of the surface properties of sporocysts in fresh, brackish and saline water and more detailed spatial modeling, including both terrestrial hosts and sea otters, will be needed to answer this question. Further insights into exposure mechanisms may come from studies of sea otter foraging behaviour in different habitats.

The observed association of high *S. neurona* seroprevalence in animals living adjacent to watersheds with high wetland area was consistent even after accounting for diet and marine habitat type. Wetlands are complex ecosystems with many features that could both positively and negatively influence the risk of infectious *S. neurona* sporocysts reaching sea otters. Firstly, wetlands are potential habitat for the definitive host of *S. neurona*, the Virginia opossum. The observed association of increased *S. neurona* seroprevalence among sea otters living near watersheds with high wetland area may reflect high definitive host density in these watersheds. Available data on opossum densities and habitat associations in North America are not adequate to determine with confidence if this is the case. Most studies on habitat associations are from the eastern USA (Table S6), and generally suggest that opossums favor human-modified environments (including developed areas and cropland) at certain times of year due to availability of anthropogenic food sources, which increase winter survival^31^. A proclivity for wetland areas is not generally noted, though some sources have indicated a preference for low-lying moist environments^32^ and streams^33^. West of the Rocky Mountains, in coastal California in particular, a markedly different climate regime means that opossums in this region are not subject to the same physiological limitations on survival imposed by cold winters in the north-eastern USA or the upper Midwest^31^, so habitat associations may differ significantly from those observed in the native range. Opossums in California have been reported to favor habitats with greater human land use intensity, small habitat fragments and proximity to urban edge areas (Table S6). Reanalysis of data from Rejmanek *et al*.^34^ showed greater success trapping opossums near habitat with open water, near developed areas and poorer success near cropping land and open grassy areas (see Supplementary Analyses and Table S7).

In addition to being potential habitat for definitive hosts, previous experimental and modeling studies have indicated that wetlands may act as filters for some terrestrial-origin pathogens. Oocysts of *T. gondii* and other similar particles appear to be trapped and retained in sediment of vegetated wetlands where they are ultimately degraded and deactivated rather than reaching the ocean^35,36^. The practical importance of this process depends on many interacting factors, not least of which is the structure of the wetland and its hydrological context. Highly canalised wetlands and those with no vegetation, little water throughput or very rapid transit time are unlikely to play a significant role in filtering infectious particles. Wetland classification for the current study was based on widely available, but imperfect data, so some misclassification (such as former wetlands that have been drained or converted to agricultural uses) may have contributed to the observed association with wetlands.

Since the only known definitive host of *S. neurona* in North America is the Virginia opossum, it is logical to expect that infection will not occur in locations far from any opossum habitat. Though little data on precise distributions and population densities is available in the literature, this species likely inhabits all areas of California included in this study, with the exception of San Nicolas Island, as well as Washington’s Olympic Peninsula. Opossums are not known to live in any part of Alaska, nor in British Columbia, except in the lower Fraser Valley and on Hornby Island^32^. Opossums have not been reported to reside on the west coast of Vancouver Island, near sea otter habitat, but the population on Hornby Island is the closest, being adjacent to the east coast of Vancouver Island. Two opossum sightings were reported in Victoria, BC, at the south-eastern tip of Vancouver Island in 1992, but none since that time^32^. As expected, low *S. neurona seroprevalence* (<10%) was recorded at all Alaskan study sites, and otters there did not have titers higher than 1:320. In contrast, the seroprevalence at the northern-most of the two Vancouver Island study sites - Nuchatlitz Inlet (BC), was 13% (4/30), and all positive animals had titers of at least 1:2560. This finding, combined with a recent report of 2 dead stranded sea otters from Vancouver Island with positive *S. neurona* PCR results^11^, indicates that infection with this pathogen is occurring in sea otters in the Vancouver Island area. The possibility of cross-reaction of the IFAT with other little-known *Sarcocystis* species occurring in the marine environment exists, but reported sequence data from the two PCR-positive stranded sea otters confirmed the presence of *S. neurona* in this region^11^. Although we cannot exclude the possibility that sea otters were infected in WA and moved to BC, such movements are rare, and infected animals were detected only in the northernmost, more remote site in BC. Therefore, the most likely explanation for our findings are that (i) opossums have colonised remote parts of Vancouver Island but remain undetected, (ii) long-distance transport of viable sporocysts is occurring (either directly or within a transport host), or (iii) a hitherto unknown (possibly marine) definitive host of *S. neurona* exists.

Long-distance transport of *S. neurona* sporocysts has not been described, but the co-occurrence of high apparent seroprevalence in Washington (both in the present study and previously reported marine mammal cases) and a predominantly westward estuarine flow out the strait of Juan de Fuca, then northwest along the coast of Vancouver Island are consistent with this possibility^37^. Two further features of this parasite necessary to render this transport process possible also exist - *S. neurona* sporocysts are highly resilient in the environment^38^ and a possible concentration mechanism has been identified in the form of bivalve filtration. Though individual-level diet data were not available for Vancouver Island sea otters, many of the animals at this location were observed to forage on the outer coast (dominated by rocky substrate) during the summer, but shift to more sheltered bays and inlets during winter. These areas are dominated by soft sediment habitat where clams are a common prey item^39^.

The seasonality of *S. neurona* infection risk is a factor that was not explored in this study, principally because it is not possible to determine how long ago a seropositive animal encountered the pathogen that elicited the antibody response. In California, a distinct pattern of *S. neurona* mortalities occurring in late spring and early summer has been identified^8^, and a strong temporal correlation has been identified between runoff events and *S. neurona* associated-mortalities of sea otters living in Estero Bay^6^. Given the seasonal nature of precipitation and river flows in California, Shapiro *et al*.^6^ attribute this seasonality in part to a ‘first flush’ effect. Sporocyst shedding by opossums also appears to peak in the spring^34^. Furthermore, seasonal variation in habitat use by opossums may exist, and habitat use in winter and spring is likely most relevant to sea otter infection risk. Future research should also focus on validating the indirect fluorescent antibody test (IFAT) in sea otters, establishing the relationship between *S. neurona* infection status and pathology and quantifying its population-level impact.

Studies at the intersection of ecology and epidemiology are enabling new uses of ecological data to clarify patterns of disease transmission, and the effects of ecosystem processes on infectious disease distribution. The sea otter has been the source of unique scientific insights into how pathogen pollutants enter the marine environment and move through marine food webs due to certain unique characteristics. Chief among these are its nearshore habitat, small home range, high trophic level and metabolic rate, and notably its tractability for observational studies - especially the fact that its unique feeding behavior allows direct quantification of individual diets^40,41^. In this study we have elucidated not only specific risk factors for exposure to one parasite, but also contributed to a more nuanced understanding of the physical processes underlying marine pathogen pollution. Our results highlight the importance of ocean physical processes in shaping spatial patterns of parasite infection risk, as well as the importance of long-term large-scale sampling in understanding these patterns. We have also shown that *S. neurona* occurs in areas outside its previously known range, raising important questions about mechanisms of parasite transmission to sea otters. Future work should seek to determine whether observed relationships with human population density, wetlands and farming are related to opossum population density or watershed modification; whether clams have a unique capability to concentrate *S. neurona* sporocysts; and, perhaps above all, whether a viable physical and biological transmission pathway exists (at least in British Columbia) on a scale not previously imagined.

## Supplementary information

Supplementary Analyses.

## Data Availability

The datasets generated during and/or analysed during the current study are available from the corresponding author on reasonable request.
