# Supplementary material for: Spatial epidemiological patterns suggest mechanisms of land-sea transmission for Sarcocystis neurona in a coastal marine mammal
Source: Sci Rep. 2020 Feb 28;10:3683. doi: 10.1038/s41598-020-60254-5 (PMC7048795; doi:10.1038/s41598-020-60254-5)
Supplement: Supplementary file 1 — Supplementary Analyses. [file 41598_2020_60254_MOESM1_ESM.pdf]

Title: Spatial epidemiological patterns suggest mechanisms of land-sea transmission for *Sarcocystis neurona* in a coastal marine mammal

Tristan L. Burgess[1,a], M. Tim Tinker[2,b], Melissa A. Miller[3], Woutrina A. Smith[4], James L. Bodkin[5], Michael J. Murray[6], Linda M. Nichol[7], Justin A. Saarinen[8], Shawn Larson[9], Joseph A. Tomoleoni[2], Patricia A. Conrad[10], Christine K. Johnson[1*]

[1]EpiCenter for Disease Dynamics, Karen C Drayer Wildlife Health Center, One Health Institute, University of California Davis, 1089 Veterinary Medicine Drive, Davis, CA 95616, USA

[2]United States Geological Survey, Western Ecological Research Center, Santa Cruz Field Station, 115 McAllister Way, Santa Cruz, CA, 95060, USA

[3]Marine Wildlife Veterinary Care and Research Center, California Department of Fish and Wildlife, 151 McAllister Way, Santa Cruz, CA, 95060, USA

[4]Department of Veterinary Medicine and Epidemiology, School of Veterinary Medicine, University of California Davis, 1089 Veterinary Medicine Drive, Davis, CA 95616, USA

[5]United States Geological Survey, Alaska Science Center, 4201 University Dr., Anchorage, AK, 99503, USA

[6]Monterey Bay Aquarium, 886 Cannery Row, Monterey, CA, 93940, USA

[7]Fisheries and Oceans Canada, Pacific Biological Station, 3190 Hammond Bay Road, Nanaimo, BC, V9T 6N7, Canada

[8]New College of Florida 5800 Bay Shore Road, Sarasota, FL 34243, USA

[9]The Seattle Aquarium, 1483 Alaskan Way, Pier 59, Seattle, WA 98101, USA

[10]Department of Pathology, Microbiology and Immunology, School of Veterinary Medicine, University of California Davis, 1089 Veterinary Medicine Drive, Davis, CA 95616, USA

[a]Current address: Acadia Wildlife Services, P.O. Box 56, South Freeport, ME, 04078, USA

[b]Current address: Nhydra Ecological Consulting, 11 Parklea Dr Head of St Margarets Bay, NS, B3Z2G6, Canada

SUPPLEMENTARY ANALYSES

Mixture model

The log-normal mixture model (Fig S1) fit the *T. gondii* serology data well, with no significant residual variation (Chi-squared goodness-of-fit test p=0.147). The results of the model were consistent with the previously determined cutoff of 1:320 - the estimated posterior positive probability of a titer of 1:320 was 0.98. A similar two-component mixture model fit the *S. neurona* serology data well (p=0.699) and showed a subjectively similar distribution to the *T. gondii* data (Fig S1). A cutoff of 1:320 was chosen, with an estimated posterior positive probability of 0.99.

Land cover data and eigenvector decomposition

Data from the 2005 North American Land Cover Database[1] (IC) were collapsed into categories for the principal land cover types represented in the dataset (See S5 table). The percentage of each land cover type was calculated by overlaying this dataset with the study watershed boundaries as previously described. Population density (persons/km^2), housing unit density (units/km^2) and road density (km/km^2) were calculated by overlaying data from the 2010 U.S. Census[2] and the 2011 Census of Canada[3] with the watershed boundaries. Eigenvector decomposition was used to address the problems created by the complex correlation structure of landuse/landcover data as well as census-derived variables. Eigenvector decomposition generated 5 orthogonal, rotated components (Table S6). Component one (C1) explained 40% of variation in the data and represented an index of human development, being composed of heavy positive loadings of human population (0.95) and housing unit density (0.95) as well as developed area (0.90), road density (0.77) and a lesser loading of row crops (0.62). Component two (C2), explaining 20% of the variation, represented a positive loading of grassland (0.96), and a negative loading on forest

area (-0.72). Component three (C3), explaining 14% of the variation, represented an index of the scrub land cover type (0.98). Component four (C4), explaining 13% of variation, correlated almost perfectly (0.96) with wetland area but was also weakly associated (0.45) with row crops. Component five (C5) explained 13% of variation and accounted principally for areas of grazing land (0.82), but was also associated with row crops (0.53).

Opossum habitat analyses

Virginia opossums (*Dildelphis virginiana*) were trapped using tomahawk live traps at 9 sites in coastal California over a total of 2550 trapping nights[4]. In order to estimate approximate associations of trapping success with habitat features, the terrestrial landcover and census variables (as used elsewhere in this paper) were calculated within a 564m radius circular buffer around each trap location (total area 100ha). This value was chosen based on the report by Gillette[5] of mean distance between most distant dens of 565m and home ranges up to 108ha. On this basis, it is likely that most of the home ranges of the trapped animals is within the buffer. Binomial regression was used to predict the success of each trap based on land cover types. Separate regression models were fit for each variable (scaled and centered) as they are highly correlated.

Sites with greatest opossum trapping success tended to be located near greater proportions of water, developed land and forest, but lower than average amounts of Grazing, grassland, croplands and other natural vegetation. Scrub and wetlands showed no significant relationship with trapping success. This small dataset should be interpreted with caution as specific habitat use is unknown, and so it is at best an indication of the general features of broad areas where opossums may be found, not an indication of actual habitat use by these animals. These caveats notwithstanding, the results that opossums may be found near water bodies, developed areas and forested areas are broadly in agreement with other studies. The negative association with agricultural land uses is contrary to previous work, but specific types of

agricultural use may vary in their importance to opossums. Additionally, proximity of agricultural lands to other important habitat components such as forest that provide den sites may be very important – most agricultural land within the study area is devoid of trees and separated from forested areas.

Literature Cited:

1. Natural Resources Canada/Canadian Center for Remote Sensing (NRCan/CCRS), United States Geological Survey (USGS), Insituto Nacional de Estadística y Geografía (INEGI), Comisión Nacional para el Conocimiento y Uso de la Biodiversidad (CONABIO) & Comisión Nacional Forestal (CONAFOR). North American Land Cover at 250 m spatial resolution. Available from: https://landcover.usgs.gov/nalcms.php. (2005).

2. US Census Bureau. TIGER Line files. Available from: http://www.census.gov/geo/maps-data/data/tiger.html. (2010).
3. Statistics Canada. Census Division Cartographic Boundary Files. Available from: http://www12.statcan.gc.ca/census-recensement/2011/geo/bound-limit/bound-limit-2011-eng.cfm. (2011).
4. Rejmanek D, Vanwormer E, Miller MA, Mazet JAK, Nichelason AE, Melli AC, Packham AE, Jessup DA, Conrad PA. Prevalence and risk factors associated with *Sarcocystis neurona* infections in opossums (*Didelphis virginiana*) from central California. Vet. Parasitol. 2009; 166:8-14.
5. Gillette LN. Movement Patterns of Radio-Tagged Opossums in Wisconsin. The American Midland Naturalist. 1980;104(1):1-12.

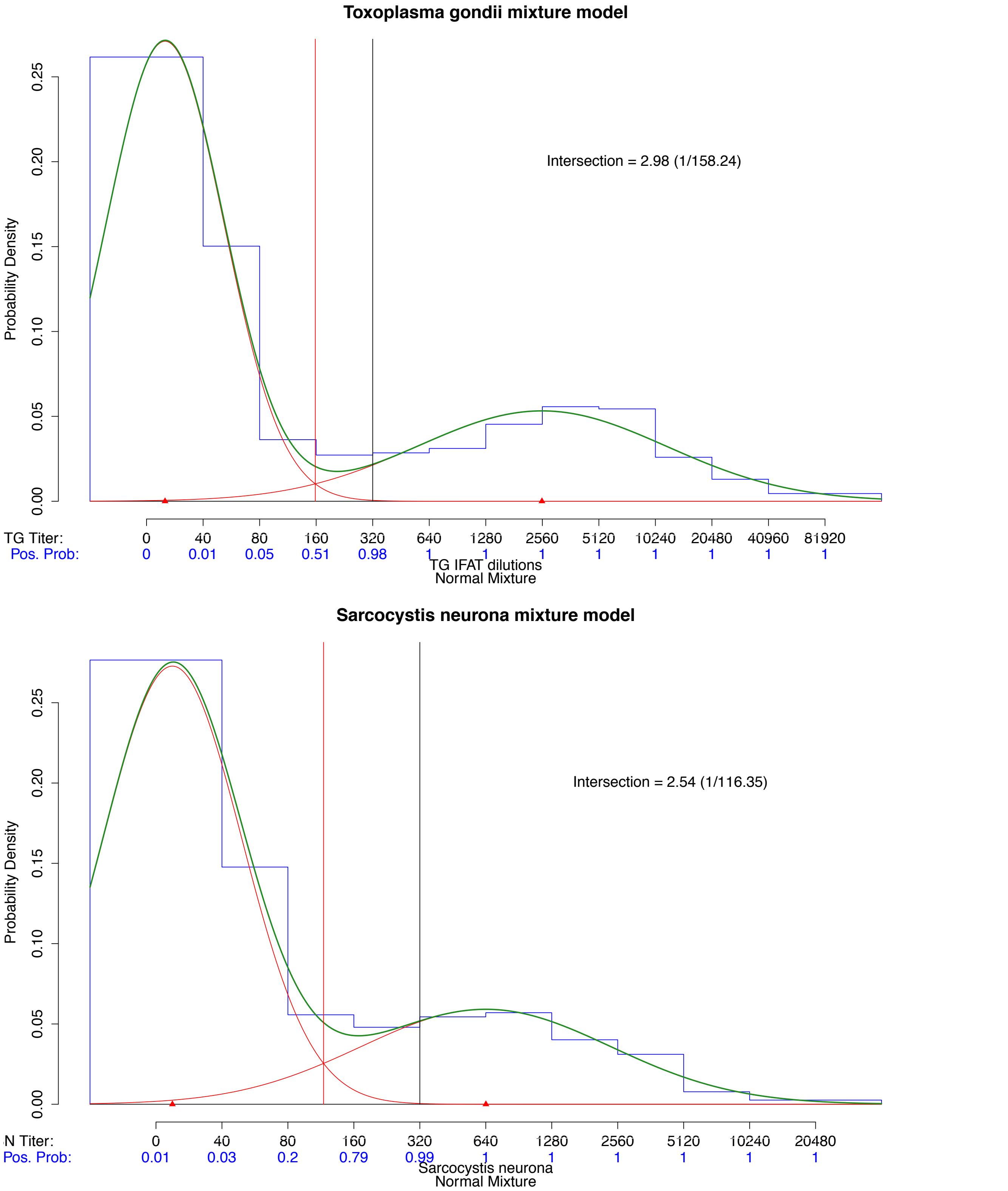

Figure S1: Mixture models. Plots of log-normal mixture models fitted to *Toxoplasma gondii* (top) and *Sarcocystis neurona* (bottom) serum indirect fluorescent antibody test (IFAT) results (n=774) from live captured sea otters (*Enhydra lutris*). The black line indicates the previously validated cutoff (*T. gondii*) or the cutoff chosen for this study (*S. neurona*). The X-axis shows the titers (black, top row) and the posterior probability a result of that titer belongs to the theoretical positive population (blue, bottom row).

Table S1: Descriptive features of watershed variables by study region.

STUDY REGIONS	CENSUS VARIABLES				LANDUSE/LANDCOVER VARIABLES							
	Area (km^2)	PD	HD	RD	Developed	Forest	Pasture	Cropping	Wetland	Other	Modified	Impervious
Western Prince William Sound, AK	1032.9	0.07	0.06	0	0.00%	33.65%	0.00%	0.00%	1.70%	64.64%	0.00%	0.00%
Elfin Cove, AK	1841.2	0.06	0.07	0.01	0.03%	48.33%	0.00%	0.00%	1.55%	50.09%	0.03%	0.00%
Whale Bay, AK	1399	0.01	0.02	0	0.00%	48.69%	0.00%	0.00%	1.25%	50.06%	0.00%	0.00%
Nuchatlitz, BC	1437.2	0.05	0.01	0	0.00%	93.14%	0.00%	0.00%	0.00%	6.86%	0.00%	0.00%
Clayoquot, BC	3833.8	0	0	0	0.00%	89.95%	0.00%	0.00%	0.02%	10.03%	0.00%	0.00%
Olympic Peninsula, WA	4704.8	1.89	0.8	1.04	1.88%	79.96%	0.19%	0.01%	2.33%	15.63%	2.07%	0.31%
Monterey Bay, CA	13374.6	63.21	21.76	2.22	9.08%	21.53%	1.30%	8.79%	1.13%	58.17%	19.17%	1.76%
Elkhorn Slough, CA	132.1	101.18	30.13	3.16	29.29%	22.01%	0.89%	7.52%	5.34%	34.96%	37.69%	4.66%
Monterey Peninsula, CA	707.2	85.96	47.94	2.24	9.40%	48.85%	0.01%	0.19%	0.52%	41.02%	9.61%	1.81%
Big Sur, CA	764.6	2.23	1.43	1.23	2.79%	59.57%	0.09%	0.16%	0.33%	37.06%	3.05%	0.21%
San Luis Obispo, CA	3675	85.89	32.83	2.15	8.90%	22.18%	1.87%	7.27%	1.16%	58.63%	18.04%	2.09%
Santa Barbara Channel, CA	375.9	0.7	0.43	1.98	5.51%	25.33%	0.01%	0.29%	0.35%	68.52%	5.80%	0.61%
San Nicholas Island, CA	69.1	0.81	0.01	0.93	6.69%	0.01%	3.46%	0.00%	0.00%	89.84%	10.15%	1.58%

PD = Population Density, HD = Housing unit density, RD = road density (US Census 2010). Percentage of developed land, forest, pasture, cropping land, wetland, other and impervious surface are calculated from the National Land Cover Dataset (USGS) and North American land cover database (NRCan/CCRS). ‘Modified’ land is any human-dominated landscape, and is calculated here as the sum of developed, pasture, and cropping land. AK = Alaska USA, BC = British Columbia Canada, WA = Washington USA, CA = California USA.

Table S2: Descriptive features of the study population. *Sarcocystis neurona* (SN) serum antibody testing results from 711 sea otters (*Enhydra lutris*) captured from 1998-2013 and watershed characteristics of the 13 study areas in the USA and Canada (BC only) where captures occurred.

STUDY REGION	YEARS SAMPLED	SEX		AGE			TOTAL
		F	M	Pup/Juv	Subadult	Adult	SN Prev.
Western Prince William Sound, AK	2010	6.3% (n=16)	0.0% (n=7)	0.0% (n=2)	0.0% (n=2)	5.3% (n=19)	4.3% (n=23)
Elfin Cove, AK	2011	0.0% (n=19)	20.0% (n=5)	-	0.0% (n=7)	5.9% (n=17)	4.2% (n=24)
Whale Bay, AK	2011	8.3% (n=24)	16.7% (n=6)	0.0% (n=1)	-	10.3% (n=29)	10.0% (n=30)
Nuchatlitz, BC	2010	11.8% (n=17)	15.4% (n=13)	-	0.0% (n=1)	13.8% (n=29)	13.3% (n=30)
Clayoquot, BC	2010	0.0% (n=11)	0.0% (n=5)	-	0.0% (n=3)	0.0% (n=13)	0.0% (n=16)
Olympic Peninsula, WA	2011	57.9% (n=19)	81.8% (n=11)	-	-	66.7% (n=30)	66.7% (n=30)
Monterey Bay, CA	1998, 2001	83.3% (n=6)	81.8% (n=11)	-	50.0% (n=2)	86.7% (n=15)	82.4% (n=17)
Elkhorn Slough, CA	2012, 2013	60.0% (n=15)	75.0% (n=8)	0.0% (n=2)	33.3% (n=3)	77.8% (n=18)	65.2% (n=23)
Monterey Peninsula, CA	1998-2013	27.5% (n=167)	35.4% (n=48)	9.5% (n=21)	0.0% (n=17)	34.5% (n=177)	29.3% (n=215)
Big Sur, CA	2003, 2008-2011	1.2% (n=82)	35.3% (n=17)	0.0% (n=5)	0.0% (n=8)	8.1% (n=86)	7.1% (n=99)
San Luis Obispo, CA	2001-2003, 2005, 2012	21.2% (n=85)	32.1% (n=56)	0.0% (n=8)	11.1% (n=18)	29.6% (n=115)	25.5% (n=141)
Santa Barbara Channel, CA	2012-2013	5.0% (n=20)	10.5% (n=19)	0.0% (n=2)	0.0% (n=2)	8.6% (n=35)	7.7% (n=39)
San Nicolas Island, CA	2003-2005	0.0% (n=10)	0.0% (n=14)	0.0% (n=2)	0.0% (n=5)	0.0% (n=17)	0.0% (n=24)

AK = Alaska USA, BC = British Columbia Canada, WA = Washington USA, CA = California USA.

Table S3: Five-dimensional eigenvector decomposition of original landuse/landcover and census-derived variables from terrestrial watersheds adjacent to sea otter (*Enhydra* lutris) habitat at the 13 study sites in Alaska (USA), British Columbia (Canada), Washington (USA) and California (USA).

**Original Variables**	**C1**	**C2**	**C3**	**C4**	**C5**
Developed area	**0.90**	0.20	0.00	0.10	0.33
Row crops	**0.62**	0.00	0.26	0.45	**0.52**
Grazing land	0.24	0.42	-0.16	0.05	**0.82**
Forest	-0.26	**-0.72**	-0.40	0.00	-0.33
Wetlands	0.24	0.03	0.00	**0.96**	0.06
Scrub	0.09	0.04	**0.98**	0.01	-0.08
Grassland	0.14	**0.96**	-0.09	0.02	0.13
Population density	**0.95**	0.07	0.08	0.19	0.17
Housing unit density	**0.95**	0.15	0.02	0.11	0.03
Road density	**0.77**	0.45	0.31	0.21	0.06
Proportion Explained	0.40	0.20	0.14	0.13	0.13
Cumulative Proportion	0.40	0.60	0.74	0.87	1.00

Table S4: Univariate logistic regression analyses predicting *Sarcocystis neurona* serum antibody status in live captured sea otters (*Enhydra lutris*) from Alaska (USA), British Columbia (Canada), Washington (USA) and California (USA) 1998-2013 (n=711).

Variable	Level	OR	SE	95% CI	P-Value
**Age**	Juvenile	1	-	-	REF
	Subadult	1.28	0.89	(0.22-7.31)	0.7804
	**Adult**	**7.52**	**0.73**	**(1.8-31.44)**	**0.0057**
**Sex**	Female	1	-	-	REF
	**Male**	**1.94**	**0.18**	**(1.35-2.78)**	**0.0003**
**Developed**	**10% increase**	**3.01**	**0.24**	**(1.87-4.83)**	**<0.0001**
**Row Crops**	**10% increase**	**6.32**	**0.26**	**(3.78-10.56)**	**<0.0001**
**Grazed Pasture**	**10% increase**	**1.36**	**0.11**	**(1.09-1.7)**	**0.0065**
Forest	10% increase	0.97	0.04	(0.9-1.04)	0.3798
**Wetland**	**1% increase**	**5.26**	**0.18**	**(3.7-7.47)**	**<0.0001**
Scrub	10% increase	0.94	0.09	(0.79-1.11)	0.4562
Grassland	10% increase	1.07	0.05	(0.97-1.18)	0.1556
Other Natural Vegetation	10% increase	1.04	0.05	(0.95-1.14)	0.3773
**Impervious Surface**	**1% increase**	**1.65**	**0.09**	**(1.39-1.96)**	**<0.0001**
**PC1 (Developed/Row Crops)**	**1 unit increase**	**1.28**	**0.09**	**(1.08-1.51)**	**0.0036**
PC2 (Grass/Negative Forest)	1 unit increase	0.79	0.11	(0.63-0.98)	0.0311
**PC3 (Scrub)**	**1 unit increase**	**0.72**	**0.11**	**(0.58-0.88)**	**0.0017**
**PC4 (Wetlands)**	**1 unit increase**	**2.34**	**0.11**	**(1.9-2.88)**	**<0.0001**
PC5 (Grazing/Row Crops)	1 unit increase	1.12	0.08	(0.95-1.32)	0.1809
**Human Population Density**	**2-fold increase**	**1.21**	**0.03**	**(1.13-1.29)**	**<0.0001**
**Human Housing Unit Density**	**2-fold increase**	**1.19**	**0.03**	**(1.12-1.26)**	**<0.0001**
**Road Density**	**2-fold increase**	**2.37**	**0.17**	**(1.68-3.33)**	**<0.0001**
**Hard Substrate**	**10% increase**	**0.73**	**0.08**	**(0.63-0.85)**	**<0.0001**
Unknown Cover	10% increase	0.88	0.12	(0.7-1.11)	0.2953
**Soft Substrate**	**10% increase**	**1.18**	**0.05**	**(1.07-1.31)**	**0.0011**
**Kelp Cover**	**10% increase**	**0.59**	**0.09**	**(0.5-0.71)**	**<0.0001**

Panel 1: sea otter individual demographic variables; *Panel 2*: Individual estimated weighted (discharge/distance) exposure to terrestrial landuse/landcover features; *Panel 3*: Individual estimated weighted (discharge/distance) exposure to first 5 components of a principal components analysis using the same terrestrial landuse/landcover features; *Panel 4*: Individual estimated weighted (discharge/distance) exposure to terrestrial human population census-derived features; *Panel 5*: Individual estimated weighted (inverse distance) exposure to marine habitat/substrate features, mainland California only (n=535).

Table S5: Quantitative diet analysis of 131 California sea otters as a percentage of biomass.

Group	Urchin	Abalone	Mussel	Clam	Snail	Crab other	Kelp crab	Cancer crab	Sand crab	Star	Worm	Chiton	Sand dollar	Octopus	Squid	Misc Rocky
Female	11.8%	12.6%	13.1%	5.8%	6.0%	5.6%	10.6%	20.4%	0.7%	1.3%	6.3%	0.9%	2.1%	0.9%	0.4%	1.4%
Male	5.5%	2.2%	9.1%	14.4%	13.4%	5.5%	4.2%	15.5%	2.9%	0.0%	17.4%	0.0%	7.7%	0.1%	1.8%	0.4%
Big Sur	9.7%	20.9%	15.8%	1.5%	6.9%	4.0%	8.6%	13.3%	1.5%	1.1%	10.7%	0.0%	2.4%	0.0%	0.0%	3.6%
Monterey Peninsula	10.9%	6.8%	11.1%	9.6%	7.5%	6.2%	9.8%	21.9%	1.0%	1.0%	7.5%	1.0%	3.4%	1.0%	0.9%	0.3%

Table S6: Summary of reported Virginia opossum (*Didelphis virginiana*) habitat use associations from published literature

Region	Positive Associations	Negative Associations	Reference
Southern California	-	Population Density (Weak)	Ordeñana et al 2010
Southern California	High landuse intensity	-	Markovchick-Nicholls et al 2008
Southern California	Small habitat fragments, urban edge	-	Crooks 2002
Indiana	Breeding/Post-breeding: Ag. fields, developed land, high road density<br>All year: Forest edge	-	Beatty et al 2013
Maryland	Low, damp wooded areas	Upland forest, open areas	Llewellyn and Dale 1964
Massachusetts	Low elevation, developed areas	Forest	Kanda et al 2006
California	Developed areas, forest, water	Grazing land, Cropping,Grasslands	Rejmanek 2009/Present study (see supplementary analyses for details)

Table S7: Univariate binary regression results predicting opossum (*Didelphis virginiana*) trapping success over 2550 trap nights at 9 sites in central California (USA). Land cover variables are derived from the national land cover database and parameterized as a scaled and centered proportion of land within a 564m radius (100ha) buffer around each trapping site.

Variable	Estimate	OR	SE	z value	p value	95%	CI	AIC
Water	0.50	1.65	0.10	4.78	0.0000	1.34	2.02	100.91
Developed	0.45	1.57	0.18	2.49	0.0128	1.10	2.23	115.42
Forest	0.29	1.33	0.13	2.15	0.0315	1.03	1.73	117.41
Scrub	0.21	1.24	0.14	1.52	0.1285	0.94	1.64	119.71
Wetlands	-0.10	0.90	0.10	-1.03	0.3013	0.74	1.10	120.55
Cropping	-0.47	0.63	0.12	-4.00	0.0001	0.50	0.79	104.04
Other natural	-0.65	0.52	0.24	-2.71	0.0066	0.33	0.83	110.48
Grass	-0.72	0.49	0.26	-2.81	0.0050	0.29	0.80	108.89
Grazing	-1.19	0.30	0.44	-2.69	0.0072	0.13	0.72	101.61
